# Making of Viral Replication Organelles by Remodeling Interior Membranes

**DOI:** 10.3390/v2112436

**Published:** 2010-11-05

**Authors:** Zsuzsanna Sasvari, Peter D. Nagy

**Affiliations:** Department of Plant Pathology, University of Kentucky, 201F Plant Science Building, Lexington, KY 40546, USA; E-Mail: zsuzsanna.sasvari@uky.edu

**Keywords:** virus replication, membrane remodeling, host factor, poliovirus, Arf1

## Abstract

Positive-stranded RNA (+RNA) viruses exploit host cell machinery by subverting host proteins and membranes and altering cellular pathways during infection. To achieve robust replication, some +RNA viruses, such as poliovirus (PV), build special intracellular compartments, called viral replication organelles. A recent work from the Altan-Bonnett laboratory [1] gave new insights into the formation of poliovirus replication organelles, which are unique subcellular structures containing many individual replication complexes as a result of dynamic cellular membrane remodeling.

Plus-stranded +RNA viruses replicate in the host cells by recruiting a set of host factors, such as proteins, membranes and metabolites. The recruited host factors then perform novel functions to promote various steps during virus replication, including assembly of viral replicase complexes (RCs) on intracellular membranes [2–8]. The outcome of the viral infection is that many original cellular processes and pathways are “rewired” during viral infections, rendering the cells dramatically different from the uninfected ones. +RNA viruses can also induce anti-viral responses by infected host cells, leading to the activation of the innate immune responses. Altogether, viruses are successful pathogens because they can reprogram host cell metabolism to support the infection process and to suppress host defense mechanisms.

After entry of the viral particles to cells, the viral +RNAs are released from the particles and translation of the viral +RNA leads to production of viral replication proteins. After the recruitment of the replication proteins and the viral +RNA to the site of replication, the assembly of RC takes place on subcellular membrane surfaces. The assembled RC first produces complementary (minus)-strand (-)RNA using the original +RNA as a template. Then, the (-)RNA is used by the viral replicase to synthesize excess amounts of new +RNA progeny, which are then released from the site of replication. The release of the new +RNA then triggers new rounds of translation and replication, formation of viral particles, or participate in cell-to-cell movement [3,9–12].

***Formation of viral replication organelles.*** One of the emerging concepts in +RNA virus replication is that some +RNA viruses assemble their individual RCs as part of large (200–400 nm) organelle-like structures, called viral replication organelles. These replication organelles include the viral RNA, viral-coded and host-coded proteins and host membranes, which are brought together by numerous interactions. The host membranes recruited for replication of different +RNA viruses are derived from various organellar membranes, such as the endoplasmatic reticulum (ER), mitochondria, vacuole, Golgi, chloroplast and peroxisome; or formed via the induction of novel cytoplasmic vesicular compartments, derived from ER or possibly autophagosomal membranes [13–18]. Thus, host membranes play crucial roles in all steps of +RNA virus replication.

A recent work from the Altan-Bonnett laboratory (Rutgers University, Newark, NJ, U.S.) gave new insights into the formation of viral replication organelles [1]. These replication organelles are the result of dynamic cellular membrane remodeling, induced by enteroviruses, such as poliovirus (PV) and coxsackievirus B3 (CVB3) [1]. PV initially starts replication on pre-existing Golgi and Trans-Golgi-network (TGN) membranes. Then, as the infection progresses, the newly made viral replication proteins are redistributed to discrete cytosolic structures, the viral replication organelles (Figure 1) [1]. The replication organelles form close to the ER exit sites and are enriched for a select group of host proteins, such as the small Ras-family GTPase Arf1; GBF1, a guanine nucleotide exchange factor (GEF) for Arf1, and PI4PKIIIß, involved in phosphatidylinositol-4-phosphate (PI4P) synthesis [1]. The likely role of GTP-bound Arf1 (the membrane-associated active form) is to recruit PI4PKIIIß and other cellular proteins. This in turn can change membrane curvature, induce transport vesicles from intracellular membrane organelles and modify the lipid composition of membranes [19,20]. It is probable that PV 3A and 3CD proteins act synergistically to disassemble the host secretory trafficking pathway and facilitate the morphogenesis of the large PI4P-rich replication organelles [1,19]. The viral replication organelles are stable structures and exist until the death of the cell. Similar replication organelles are likely also formed during flavivirus infections [1].

Coronaviruses, such as SARS, also form similar intracellular structures with double-membrane vesicles and convoluted membranes, which are interconnected [21]. However, the formation of the coronavirus-induced replication organelles are different from PV as they exploit EDEMosomes that are involved in ERAD tuning, a regulatory pathway of ER-associated protein degradation (ERAD) [22].

***Role of lipid factors in the assembly of the viral replication complex.*** Another major finding by Hsu *et al.* [1], is that the viral replication organelles not only consist of hijacked cellular vesicles decorated by viral proteins, but these vesicles have also altered lipid composition. This is due to the selective recruitment of PI4PKIIIß, which leads to the production of phosphatidylinositol-4-phosphate (PI4P). In uninfected cells, PI4PKIIIß is recruited and activated by Arf1 on the Golgi and TGN membranes. However, in enterovirus infected cells, the viral 3A replication protein could be responsible for the selective recruitment of PI4PKIIIß, which was co-purified with the viral replicase complex [1]. The presence of PI4PKIIIß in the viral replication organelle leads to the enrichment of the membrane-compartment with PI4P (Figure 1) [1], which, in turn, helps the assembly of the viral replicase due to efficient binding of the enterovirus 3D^pol^ to PI4P in the membrane. The presence of high local concentration of PI4P might help selective recruitment and activation of host proteins, promote high local concentration of host factors and viral proteins as well as it affects membrane curvature, which is important to create local membrane invaginations to shield the viral replicase and viral RNA from host defense.

The PV replicase assembled on the Golgi/TGN membrane contains poly(C)-binding protein (PCBP) and the virally coded 3CD bound to the 5’ cloverleaf-like structure, while poly(A)-binding protein (PABP) binds to the 3’ poly(A) tail of the viral +RNA (Figure 1D). Due to protein–protein interaction, the ends of the PV RNA genome are brought into proximity (genome circularization), facilitating the cleavage of 3CD and the release of 3D^pol^ [23–26]. The 3D^pol^ protein then starts minus-stranded RNA synthesis using Vpg-UU as a primer [27]. This is then followed by +RNA synthesis, which will produce excess amounts of +RNA progeny. Altogether, the PI4P-rich lipid microenvironment is essential for RNA synthesis by 3D^pol^ during PV replication [1].

***Disruption of the conventional secretory pathway by enteroviruses.*** The formation of the viral replication organelles changes the subcellular distribution of key trafficking proteins, such as COPI coat protein, clathrin and γ-adaptin, to the cytosol from the typical ERGIC and Golgi locations [1]. This inhibits the vesicular trafficking pathway between the ER and Golgi, resulting in disassembly of the Golgi apparatus and disruption of the secretory pathway. Subversion of the cellular secretory pathway by enteroviruses for replication is not detrimental for enterovirus infection since the secretory pathway does not affect the production or release of the viral progeny. Moreover, disruption of the secretory pathway by enteroviruses via decoupling Arf1 activity from COPI recruitment is important for viral suppression of immune responses.

***Future directions.*** In spite of the major advances in our understanding of the role of membrane morphogenesis in PV replication [1], many questions still remain. For example, the PV replication organelles consist of nonuniform vesicles of different shapes and sizes from 70 to 400 nm. These vesicles have either single- or double-membranes, and also altered lipid composition, but their actual roles in PV replication have not yet been dissected [28,29]. The PV replicase can form planar and oligomeric arrays, called lattices [30], but how these two-dimensional protein arrays function on the surfaces of PV-induced vesicles is still uncertain. Also, the *in vivo* roles of rosette-like structures visualized after extraction, which contain the PV replication proteins, and the double-membrane vesicles are not yet understood [29]. Moreover, the PV-induced double-membrane vesicles resemble structures generated by authophagy, and are possibly involved in viral replication, virion assembly, maturation, virus export and egress [31,32]. The direct evidence supporting the connection between PV replication organelles and autophagy, however, is still missing.

The functions of the recruited host proteins within the viral replication organelles and their complete interactions with the viral replication proteins have not yet been fully analyzed. It seems that the Sec7 domain of GBF1, which has the GEF function for Arf1, is not critical for PV replication [33]. However, PV replication is dependent on the N-terminal region of GBF1, which is involved in protein interactions, suggesting new roles for GBF1. It will also be interesting to investigate the mechanism leading to the cytosolic relocalization of COPI and p115 membrane protein (important for GBF1 binding to the membrane) in PV infected cells [33].

## Figures and Tables

**Figure 1.**
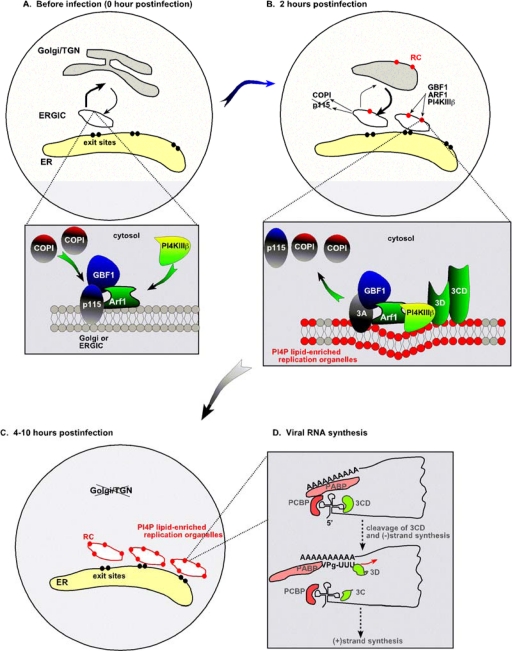
**Remodeling the cellular secretory pathway to support enterovirus replication. (A)** Trafficking between the ER and the Golgi is shown schematically in uninfected cells. The ER-Golgi intermediate compartment (ERGIC) is decorated with COPI, which is recruited by the small GTPase Arf1 and the guanosine exchange factor GBF1. **(B)** At the early time point, enteroviruses, such as PV or CVB3, starts replication in Golgi/TGN compartment. The virally-coded 3A tail-anchored membrane protein binds to GBF1 and Arf1 and promotes the redistribution of COPI and p115 membrane protein to the cytosol. These events inhibit the vesicular transport between the ER and Golgi/TGN. **(C)** At 4–10-hour time points of infection, synthesis of newly made abundant 3A molecules leads to remodeling of the Golgi/TGN and ERGIC compartments into viral organelles. The replication organelles, indicated by red ovals, contain increased amount of PI4P lipids due to the recruitment of PI4KIIIß, which makes PI4P, via Arf1 with the help of 3A. The enriched PI4P contents of these replication organelles facilitate the binding of 3D^pol^ or 3CD to the membrane, thus promoting the assembly of the replicase complex (RC) and viral RNA synthesis. The figure was modified from [1]. **(D)** Factors involved in the RNA synthesis by the PV replicase assembled on the Golgi/TGN membrane.
